# Reducing widespread pipe sharing and risky sex among crystal methamphetamine smokers in Toronto: do safer smoking kits have a potential role to play?

**DOI:** 10.1186/1477-7517-9-9

**Published:** 2012-02-16

**Authors:** Charlotte Hunter, Carol Strike, Lorraine Barnaby, Adam Busch, Chantel Marshall, Susan Shepherd, Shaun Hopkins

**Affiliations:** 1 Human Biology Program, University of Toronto, Toronto, Canada; 2 Dalla Lana School of Public Health, University of Toronto; Centre for Addiction and Mental Health, Toronto, Canada; 3 Shout Clinic, Central Toronto Community Health Centres, Toronto, Canada; 4 AIDS Committee of Toronto, Toronto, Canada; 5 The Works - Needle Exchange, Toronto Public Health, Toronto, Canada; 6 Toronto Drug Strategy Secretariat, Toronto Public Health, Toronto, Canada; 7 Dalla Lana School of Public Health, University of Toronto, 155 College Street, Toronto, ON M5T 3M7, Canada

**Keywords:** Crystal methamphetamine, Qualitative, Harm reduction

## Abstract

**Background:**

Crystal methamphetamine smoking is associated with many negative health consequences, including the potential for transmission of hepatitis. We examined whether or not a kit for crystal methamphetamine smoking might have some potential to reduce the negative health effects of crystal methamphetamine smoking.

**Methods:**

Five focus groups were conducted with crystal methamphetamine smokers recruited by community health agencies and youth shelters in Toronto, Canada. Target groups included homeless/street-involved youth, sex workers, men who have sex with men, and youth in the party scene. Participants (n = 32) were asked questions about motivations for crystal methamphetamine use, the process of smoking, health problems experienced, sharing behaviour, risky sexual practices, and the ideal contents of a harm reduction kit.

**Results:**

Pipe sharing was widespread among participants and was deemed integral to the social experience of smoking crystal methamphetamine. Heated pipes were unlikely to cause direct injuries, but participants mentioned having dry, cracked lips, which may be a vector for disease transmission. Many reported having sex with multiple partners and being less likely to use condoms while on the drug. Demand for harm reduction kits was mixed.

**Conclusions:**

Changing pipe sharing behaviours may be difficult because many participants considered sharing to be integral to the social experience of smoking crystal methamphetamine. Within the context of a broader health promotion and prevention program, pilot testing of safer smoking kits to initiate discussion and education on the risks associated with sharing pipes and unprotected sex for some communities (e.g., homeless/street-involved youth) is worth pursuing.

## Background

Crystal methamphetamine smoking is associated with many negative health consequences and is linked with transmission of Hepatitis C virus (HCV) [1]. Heated and damaged pipes may lead to injuries to the lips and mouth [1] and when shared these pipes may be a vector for Hepatitis C virus (HCV) transmission. A systematic review concluded an HCV prevalence ranging from 2.3 to 5.3% among never-injecting drug users represents a serious health concern among this population but the causal mechanism of transmission was unclear [2]. Populations most often associated with smoking crystal methamphetamine include homeless/street-involved youth, gay men, sex workers, and youth in the party scene [1].

Crystal methamphetamine smoking has also been linked with risky sexual behaviours. Studies show crystal methamphetamine increases sex drive and can enable longer sexual episodes; it also leads to drying of the mucosa, which can cause tears in the genital region and facilitate transmission of HIV or other sexually transmitted infections [3]. A study of sexually active adults in California found that non-injection methamphetamine use was inversely associated with condom use, regardless of the type of intercourse [4]. Low rates of condom use (one third of the time during vaginal sex and one quarter of the time during anal sex) in another study lend support to this finding [5]. Greater intensity of methamphetamine use was also positively associated with unprotected sex in a sample of 261 HIV-positive MSM in California [6].

Like other drug use related problems, options to reduce negative consequences span the four pillars of drug policy from prevention to drug treatment to law enforcement to harm reduction. In terms of harm reduction, transferring the model of safer injection kits is one option and holds some appeal. An abundance of evidence demonstrates the effectiveness of needle and syringe programs which are considered as an essential component of an HIV prevention program [7]. The equipment distribution model has been transferred to address concerns surrounding crack cocaine smoking [8-10]. Typically, crack smoking kits contain a glass stem, a screen, a push stick and a mouth piece [8-10]. However, the evidence regarding safer crack kits as a means of preventing disease transmission is less definitive [9,11-16] than it is for needle distribution. While the evidence is not definitive, the rationale for this type of intervention holds true for crystal methamphetamine smoking which shares many of the risks associated with smoking crack cocaine. However, the design of the stem used in crack kits is not ideal for smoking crystal methamphetamine, which liquefies when heated and may be inhaled if smoked with a glass stem, and alternative designs would need to be explored.

In our study, we examined whether or not a kit for safer crystal methamphetamine smoking might have some potential to reduce the negative health effects of this method of drug use.

The design of a harm reduction programs such as safer smoker kit, and other public health interventions, requires an understanding of the target population; underlying behavioural motivations; behavioural patterns; social context of behaviours; perceptions of harm and susceptibility; perceived benefits and barriers; kit preferences and perceived willingness and ability to modify behavior [17,18]. Ultimately, our goal was to answer the question: is a crystal methamphetamine kit a desirable harm reduction tool, and if yes, what might a kit contain?

## Methods

Between January and March, 2011, we conducted focus group discussions at community agencies in Toronto that served homeless/street-involved youth, gay men, sex workers, and youth who are not homeless/street involved but are involved in a party scene where drugs such as crystal methamphetamine are commonly used. Focus groups were selected because they are an efficient method of data collection and provide the opportunity to examine consistency and difference in opinions and experience within a group. Using poster advertisements and word of mouth, we asked each agency to recruit clients who had smoked crystal methamphetamine in the past month for the study. Focus groups were co-moderated by one of the lead researchers and another research team member. Participants were asked to provide verbal consent and received $25 and two transit tokens for participation. This project was approved by the Office of Research Ethics at the University of Toronto.

During the discussions, participants were asked about factors that lead individuals to smoke crystal methamphetamine; the process of smoking crystal methamphetamine; the types of equipment used for smoking; the frequency of equipment sharing; recommendations for the contents of an ideal 'safer crystal methamphetamine smoking' kit; and health consequences of smoking crystal methamphetamine. Each participant completed a short demographic questionnaire.

The focus groups were audio-recorded. The audio-recordings were reviewed and detailed notes compiled. The notes for each focus group were reviewed by both moderators and revised as necessary. These notes were managed using a word processing package. We followed an iterative analytic procedure [19]. Using the discussion guide questions as an initial structure, the notes were analysed for key themes and a coding structure created. Team members met to discuss and revise the coding structure. Thematic memos were written to describe, summarize, and analyse the content of each theme. Supporting illustrative quotes were identified in the audio-recordings and added to each memo. The recordings were reviewed periodically to ensure the accuracy of the memos. We compared thematic content across and within focus group discussion. All team members reviewed and revised the final analyses to ensure accuracy and completeness.

## Results

Table 1 describes the characteristics of the participants who attended one of five focus groups.

**Table 1:** Demographic characteristics and self-reported drug use

Demographic factor	Number (%)
Gender	
Male	22 (68.8)
Female	7 (21.9)
Transgender male to female	3 (9.4)
Age	
15 to 19 years old	6 (18.8)
20 to 29 years old	21 (65.6)
30 to 39 years old	2 (6.2)
40 years or more	3 (9.4)
Born in Canada	
Yes	29 (90.6)
No	3 (9.4)
Ethnicity (list all that apply)	
White (Caucasian)	21 (65.6)
Black	3 (9.4)
First Nations/Inuit/Métis	5 (15.6)
Other	3 (9.4)
Left blank	3 (9.4)
Non-injection drugs used in past year (in addition to methamphetamine)	
Cocaine or crack cocaine	23 (71.9)
Heroin or other opiate	10 (31.2)
Ecstasy, Ketamine, or other club drug	27 (84.4)
Other	14 (43.8)
Poly drug user (non-injection)	30 (93.8)
Injection drugs used in the past year	
None	13 (40.6)
Cocaine or crack cocaine	6 (18.8)
Heroin or other opiate	6 (18.8)
Methamphetamine	14 (43.8)
Speedball	5 (15.6)
Ketamine	9 (28.1)
Other	3 (9.4)
Poly drug user (injection)	10 (31.2)

### Motivations for using crystal meth

When describing their use of crystal methamphetamine, participants described varied reasons for using the drug: pharmacological, physical, psychological, emotional, cognitive and sexual. Commonly discussed was the big rush of adrenaline and feeling of euphoria that accompanies crystal methamphetamine smoking. Across all groups, participants talked about feeling a boost in energy after smoking it and the ability to use for extended periods of time without sleep. Delaying sleep helped those who worked at night. However, more often participants talked about how increases in energy helped them enjoy all night dance parties. Also, the boost in energy led many to feel more proactive and productive. Amongst the participants, only one mentioned smoking methamphetamine because of feeling addicted to it.

*Right away you just feel amazing, and you feel like you can do anything. You have a lot of energy. Pretty much everything in your mind is going right... You're just in an amazing world, pretty much*.

*I have too many things to do when I'm on it to bother with eating or sleeping. I'm on the go, I want to do stuff*.

As well as providing a boost in energy, participants also discussed how smoking crystal methamphetamine improved their self-esteem, confidence and sociability. Crystal methamphetamine smoking was also linked with improvements in clarity of thought, concentration and ability to study 'e*specially in college or university, come exam time, when you have to do all that cramming.'*For a small number, smoking crystal methamphetamine increased their creativity and artistic productivity. A participant remarked '*It helps me be more artistic. I find, like, I can draw and paint better.' *It helped many to manage mood swings and reduce feelings of depression and other unwanted emotions.

*With me, like, I have a lot of trauma in my past. Like, I used to be a cutter. I have suicide attempts under my belt, and so when I get into those moods, instead of harming myself or harming others I just smoke some crystal and it just goes away*.

For some participants, the effects of crystal methamphetamine helped them to overcome self-stigma and negative feelings about being gay. For those involved in sex work, combined effects of increased awake time, energy and confidence with reduced negative emotions were desirable.

Across all discussion groups, participants strongly endorsed the positive effect of smoking crystal methamphetamine on sex. It made them feel sexier. While using methamphetamine, sex was described as better in terms of increasing its duration and physical intensity. Some participants described the increased sensitivity to physical touch. With few exceptions, participants described how they felt more sexually adventurous and less inhibited while on the drug. Gay men described feeling sexier, less worried about being rejected and less concerned that they could not perform as well as they might want.

*I'm kind of sexually inhibited, so it allows me to access part of myself that I normally can't... It kind of allows me to get past those fears of rejection, or fears of not being good enough for somebody or not being sexy enough for somebody, because it makes you feel sexy*.

During some discussions, participants described smoking crystal methamphetamine as a weight loss strategy. Amongst participants experiencing housing and food insecurity, smoking crystal methamphetamine reduced their appetites and feelings of hunger.

### Smoking and equipment types

Basically, you put your crystal into [the pipe] and you should keep the lighter about an inch below the bottom of the pipe. You let it melt into a liquid form and you wait a second until it re-crystalizes over, and then you heat it up until it puddles again. You keep it constantly moving while inhaling not as hard as you would with crack, but not slow, just like a normal sized breath

When asking about the process of smoking, the most common ways described involved the use of a store-bought ball pipes (a glass stem with a bowl attached) or using tin foil and straws, a method known as "chasing the dragon". With the exception of participants who were homeless/street-involved, few discussed the use of improvised equipment such light bulbs, soft drink cans and/or ginseng vials. For the homeless participants, improvised pipes were only used when ball pipes were not available. Some participants had used crack stems to smoke crystal methamphetamine or had heard of others doing so. Smoking with a crack stem was generally considered an unsuitable method because it does not have a bowl to collect the liquefied crystal methamphetamine that prevents this liquid from being inhaled and/or swallowed. During several groups, a technique called "hot-railing" was described. Hot railing involves heating a crack stem and then inhaling a line of vaporized crystal methamphetamine through the nose. Although uncommon, a few participants mentioned converting crack stems into ball-type pipes. Some had seen friends or acquaintances heat a crack stem and then 'blow' out a ball that would be used to collect and smoke liquefied crystal methamphetamine. After hearing about this technique, we asked other participants but few were familiar with it.

Smoking with a ball pipe, especially one made of Pyrex, was considered by the participants to be the safest way to smoke crystal methamphetamine. This method involves putting the crystals in the bowl of the pipe and using a torch lighter to heat the bowl from below but keeping it an inch away. The crystal methamphetamine turns to liquid and then to vapour, which the user inhales. Frequency of use varied as much within groups as between groups, ranging from every day to every few weeks or months. Participants in several groups described having been on binges where they smoked every day for several weeks or even a whole month, during which they were awake almost the entire time.

### Health problems experienced

Alongside the positive benefits of smoking crystal methamphetamine was a wide array of negative health problems. The most commonly described included: dry mouth and dehydration, poor nutrition, fatigue, sleep deprivation, psychological or perceptual problems (e.g., depression, paranoia, and auditory or visual hallucinations), skin problems, including blemishes, and bowel irregularities.

*I pretty much stayed up for like two and a half weeks so it was hard to get to sleep. It was hard to sleep, you know, I was trying this and that and I wasn't eating properly... I didn't look really healthy. Things were coming out of my face. I felt really tired and I started losing more and more weight*.

In response to specific questions about the impact of crystal methamphetamine on their oral health, very few participants mentioned problems with their teeth, gums and/or the tissues in and around their lips and mouths. None of the groups raised chapped lips as a health concern, however, three of the groups suggested that lip balm be included in the kits.

When asked about injuries and burns to hands or lips from touching hot pipes, most participants said that this was rare because they used only the bowl-type pipes.

*I've never seen it happen... I think the reason [why it happens with crack and not crystal] is, because with crack, the heat is right on the stem, where we heat the bowl, and you have to heat it a lot more with crack... Because you hold the stem partway down with crystal, it would never burn your lips cause you'd burn your fingers first. It just doesn't happen*.

Injuries and burns were linked more to smoking crack cocaine or to the use of improvised pipes (e.g., light bulbs) for smoking crystal methamphetamine. Participants who had smoked crack cocaine said less heat was required to vaporize and inhale crystal methamphetamine than to burn crack cocaine to smoke. Reduced heating was linked with fewer burns to the hands, lips and mouth. The participants clarified further that crystal methamphetamine is vaporized, not burned, and that if you used the amount of heat applied to crack pipes with a crystal methamphetamine pipe you would burn the drug. In general, cuts and burns from smoking with any device was deemed to be an issue related more or less to personal skill.

### Sharing behaviour

*It could be the whole party, it could be two people onto one pipe, it could be five people, it could be everyone... It depends on who has a pipe and who doesn't*.

With only one exception, participants in all focus group discussions declared that pipe sharing was ubiquitous amongst people who smoke crystal methamphetamine. Participants noted that at some parties or bathhouses only one or two pipes might be available and all present share the pipe and the drugs. The exception was the group from the party scene program, where most participants were more protective of their pipes and felt they might only share with one or two close friends. Generally, however, most participants spoke with little to no concern about any potential negative health outcomes from sharing crystal methamphetamine pipes and/or other devices. Amongst the minority of participants who discussed crystal methamphetamine within the context of sex and sex work within bathhouses, many noted that the drug and the pipes are shared in exchange for sex. When concerns about sharing pipes were raised, most often these were in relation to worry about someone breaking a pipe, smoking more than their 'share' of the crystal or burning and wasting the crystal methamphetamine, rather than about the possibility of disease transmission. Some participants felt that sharing with others at a party was automatic. When asked more directly about any concerns related to sharing pipes, a few participants noted that they were more likely to share with people who they knew well because they would know if the person had a disease or not. Pipes used were rarely brand new, and most people would only get a new pipe if their old one broke, regardless of how many people had used it in the past.

### Safer crystal methamphetamine smoking kit

When asked about the ideal pipe to be included in a kit for safer crystal methamphetamine smoking, most participants agreed that tempered glass or Pyrex ball pipes were the best and the least likely to break. While many believed that longer stems were safer, there was considerable variation regarding preferred stem length. Some preferred shorter stems that are easier to carry and conceal whereas others liked longer stems that increased the distance from the heat source to face and hands. The size of the bowl and its ventilation hole were considered more important than the length of the stem. The bowls need to be sufficiently large to hold liquefied crystal methamphetamine and the hole sufficiently large to allow oxygen into the bowl for vaporization. Neither could be too big (i.e., difficult to carry and conceal) or too small (i.e., insufficient room for the liquefied crystal methamphetamine or insufficient oxygen necessary for vaporization).

Beyond the most important piece of equipment, the pipe, a wide variety of contents were suggested for a 'safer crystal methamphetamine smoking' kit (see Table 2). Suggested locations to distribute the kits varied from those open during business hours to those open or available after hours: community health agencies, youth shelters, mobile health buses, bathhouses and dance venues or clubs. Several participants said that bathhouses and clubs are good locations but that owners intent on keeping drug use hidden might resist distribution of the kits on site.

**Table 2:** Suggested contents of a 'safer crystal methamphetamine smoking' kit

Item	Reasons and comments
• 1-2 Pyrex or tempered glass pipes • Lighter • Scoops • Scrappers • Alcohol wipes	• To prevent breakage • Torch lighters preferred • To put crystal meth in bowl • To scrape out residue • To clean pipe after use • For "chasing the dragon"
• Tin foil and straws • Hand sanitizer • Condoms	
• Lubricant • Mouthwash	• For oral hygiene concerns • For cracked lips
• Lip balm	
• Band-Aids • Rubber mouthpieces • Gum • Electrolyte powder • Educational pamphlet	• Many people would not use • For dry mouth • Since not eating much • With information about health risks, crisis phone numbers, etc.

While participants were forthcoming with suggestions for the contents of the kits, there was considerable variation across the groups about the perceived demand for and/or desirability of the kits amongst their population group. The perceived demand was highest for the homeless/street-involved youth without the means of purchasing pipes. Gay men and party scene goers said that they would take free kits but if obtaining them was inconvenient they would be more likely to buy their own pipes.

When asked if the distribution of kits might reduce pipe sharing, most participants expressed doubt. Gay men and the homeless/street-involved youth felt that the social aspect of sharing pipes was an important driver of crystal methamphetamine use, as part of social gatherings and in the sexual transactions occurring inside and outside of the bathhouse scene.

No... There's a social element to crystal meth... One person's using it and that's the bait that attracts everyone else... You can be the centre of attention

Crystal methamphetamine is difficult to divide into discrete portions; consequently, sharing is common amongst those who have contributed to its purchase, are generously sharing and/or are exchanging some crystal methamphetamine for sex, money or other benefits.

### Sexual risk taking

As noted above, crystal methamphetamine smoking is linked with perceptions of better, longer and more adventurous sex. It is also linked with sex with multiple partners. Furthermore, several participants said that sex acts could be rougher and more potentially damaging while on crystal methamphetamine. When asked, some participants said they were less likely to use condoms when smoking crystal methamphetamine. However, the frequency of condom use was said to be related to a person's overall attitude towards condoms.

*It's like showering with a raincoat on. You can't feel the increase in sensitivity from skin contact*.

Those who disliked using condoms reported they smoked crystal methamphetamine to give them "permission" not to wear a condom during sex. This was particularly true for some gay men who participated in our study. Given the low rates of reported condom use, we asked if it was beneficial to include a condom in a safer smoking kit. Several participants said recipients might be more inclined to use one if it was included in the kit. One individual offered that he might hand a condom from the kit to a sex partner if he "had a vibe about him" or "looked kind of dirty".

## Discussion

Our data show that pipe sharing was common and widespread among all groups of crystal methamphetamine smokers in this study. It was viewed as a normal part of the culture of smoking this drug, which is often done in a group setting. The majority of participants in the consultations were not concerned with and/or unaware of the potential health risks from sharing pipes primarily because they viewed the risks from sharing pipes as trivial in comparison to the risks associated with unprotected sex. Sharing was described as a typical feature of the smoking experience, both for practical reasons, including not wanting to split a quantity of the drug between several pipes, and for social reasons. Most participants connected sharing to parties, dancing at clubs, "sex parties" in the gay community and sexual encounters at bathhouses. In this way, some of the drivers of crystal methamphetamine smoking also drive the sharing behaviour.

While we obtained valuable information about the ideal contents of a harm reduction kit for crystal methamphetamine smoking, our data lead us to question if the kits might be used at all and/or used for the intended purpose of reducing sharing. With the exception of homeless/street-involved youth, many participants were hesitant to say that a safer crystal methamphetamine smoking kit would lead to changes in their behaviour. Crystal methamphetamine is often smoked in a group setting where sharing is a part of the culture of smoking and not the result of an inability to buy or access new and clean supplies. Questions about ease of purchase revealed that it is relatively easy to purchase a suitable pipe. Research team members had no difficulty purchasing pipes to show during the focus group discussions. Nevertheless, there were a minority of homeless/street-involved participants who lacked sufficient resources to purchase a pipe. Data from a 2009 Toronto study amongst street youth (n = 100) showed that 74% youth rated access to a safer crystal meth kit as high on their demands [20]. Among youth in that study who smoked crystal methamphetamine, 83% used a glass pipe with a bowl, 40% used a homemade pipe made from a light bulb, 21% smoked it using tin foil, 19% used a crack pipe and 8% used a metal pipe. While our findings related to sharing behaviour lead us to question whether or not kits would decrease sharing amongst this population, access to kits might reduce the use of improvised equipment (e.g., light bulbs) said to be more likely to cause injury and burns. Amongst all participants, gay men were the least convinced that the kits would reduce sharing at parties because the social aspect of sharing a pipe was an important part of the experience and integral to the sexual transactions occurring in bathhouses. They also felt that the risk of disease transmission associated with pipe sharing was trivial in the context of the unprotected sex occurring in settings where crystal methamphetamine was used. Future studies targeting crystal methamphetamine smokers should examine more thoroughly whether harm reduction services could actually reduce pipe sharing.

Another striking finding was the insistence by almost all groups that injuries to the mouth (e.g., cuts and burns) and tooth decay (i.e., meth mouth) were not as much of a concern for crystal methamphetamine smokers as popularized in the media [21]. However, amongst crystal methamphetamine smokers in the Shout Clinic study, 35% reported cracked lips, 35% burns and cuts to hands and 18% burns and cuts to the lips [20]. While findings are mixed, current research suggests that poverty, homelessness, personal hygiene and drug-related effects (e.g., reduced salivation; teeth-grinding) are key contributors to the oral health status of this group of drug users as opposed to the use of crystal methamphetamine [22-26]. However, the recommendation by many study participants to include lip balm in the harm reduction kits suggests that dry, cracked lips associated with smoking the drug might still provide a route of entry for Hepatitis C and other blood-borne infections. Given the frequency of sharing and the prevalence of this health problem, more information is needed about the potential for disease transmission via pipes use to smoke crystal methamphetamine. In spite of these uncertainties, using crystal methamphetamine harm reduction kits as a way to make contact with drug users in need of health services or to disseminate public health information is an option worth considering. Safer crack use kits have been used in this way to reach the most isolated or marginalised drug users [27,28].

Some notions about personal risk and assumptions about the disease status of others that emerged in the focus groups were troubling. Existing clients from local AIDS service organisations involved with this study were likely exposed to extensive education about preventing the spread of HIV and other STIs. Generally, these participants were unconcerned about consistent condom use and some relied on a sexual partner's physical appearance to determine likelihood of being infected. This may be a common finding within this community, as a previous study found that when making assumptions about the serostatus of a sex partner, HIV-positive MSM based 25% of their assumptions of a negative serostatus and 3% of their assumptions of a positive serostatus on physical appearance [29]. On a similar note, other participants in our study believed that having known someone for a long time or trusting someone based on their appearance were sufficient criteria for pipe sharing, even if they were aware of the risk of Hepatitis C transmission. These findings point to a need to re-examine the effectiveness of safer sex campaigns for crystal methamphetamine smokers.

One limitation of this study was that participants were all existing clients of community health agencies or youth shelters in Toronto, and the experiences of the most marginalised or isolated crystal methamphetamine smokers may not be well represented. In addition, our analysis could have been furthered if information was collected on length and frequency of use. On the whole, however, the exploratory nature of this study allowed us to obtain valuable information about the social context of crystal methamphetamine smoking in Toronto, the wide range of associated health concerns, and suggestions for future interventions.

## Conclusion

Our findings and the design of our study prohibit a definitive statement regarding how to proceed with harm reduction programming for people who smoke crystal methamphetamine. Changing pipe sharing behaviours may be difficult because many participants considered sharing to be integral to the social experience of smoking crystal methamphetamine. However, our findings do suggest the need for a broad health promotion and prevention program for people who smoke crystal methamphetamine. Pilot testing of safer smoking kits as part of a safer smoking program to initiate discussion and education on the risks associated with sharing pipes and unprotected sex for some communities (e.g., homeless/street-involved youth) is worth pursuing. A series of semi-structured interviews with crystal methamphetamine-using street youth in Vancouver revealed that participants were managing their mental health problems with the drug rather than accessing mental health services [30]. There is also evidence to show that street youth may use crystal methamphetamine to cope with food insecurity [30,31]. A broad health promotion and prevention program might include pilot testing of a safer smoking kit and also a safer sex education campaign, mental health services, housing supports, and/or nutrition programs.

## Abbreviations

HCV: Hepatitis C.

## Competing interests

The authors declare that they have no competing interests.

## Authors' contributions

All authors contributed to the design of this study and the interpretation of the results. CH, CS and CM moderated the focus groups. CH conducted the preliminary analyses of the data. CH, CS, LB, CB, AB, SH, SS all contributed to the final analyses of the data. All authors read and approved the final manuscript.
